# Tetra­kis(μ-acetato-κ^2^ *O*:*O*′)-bis­[(3-pyridine­carboxaldehyde-κ*N*′)]dicopper(II)(*Cu—Cu*)

**DOI:** 10.1107/S1600536812041074

**Published:** 2012-10-10

**Authors:** Adriana Cruz-Enriquez, Alberto Baez-Castro, Herbert Höpfl, Miguel Parra-Hake, Jose J. Campos-Gaxiola

**Affiliations:** a Facultad de Ingenieria Mochis, Universidad Autonoma de Sinaloa, Fuente Poseidon y Prol. A. Flores S/N, CP 81223, C.U. Los Mochis, Sinaloa, Mexico; b Centro de Investigaciones Quimicas, Universidad Autonoma del Estado de Morelos, Av. Universidad 1001, CP 62210, Cuernavaca, Morelos, Mexico; c Centro de Graduados del Instituto Tecnologico de Tijuana, Blvd. Industrial S/N Col. Otay, CP 22500, Tijuana, B.C., Mexico

## Abstract

The binuclear title compound, [Cu_2_(CH_3_CO_2_)_4_(C_6_H_5_NO)], is located about a center of inversion. The Cu^II^ atoms are connected [Cu—Cu = 2.6134 (5) Å] and bridged by four acetate ligands. Their distorted octa­hedral coordination geometry is completed by a terminal pyridine N atom of a 3-pyridincarboxaldehyde ligand. In the crystal, the complex mol­ecules are linked by C—H⋯O hydrogen bonds, forming two-dimensional networks lying parallel to the *ab* plane. These networks are linked *via* C—H⋯O hydrogen bonds involving inversion-related 3-pyridinecarboxaldehyde ligands, forming a three dimensional supra­molecular architecture.

## Related literature

For related paddle-wheel structures, see: Aakeröy *et al.* (2003 ▶); Sieroń (2004 ▶); Fairuz *et al.* (2011 ▶); Trivedi *et al.* (2011 ▶). For Cu⋯Cu separations in related structures, see: Seco *et al.* (2004 ▶); Asem *et al.* (2011 ▶).
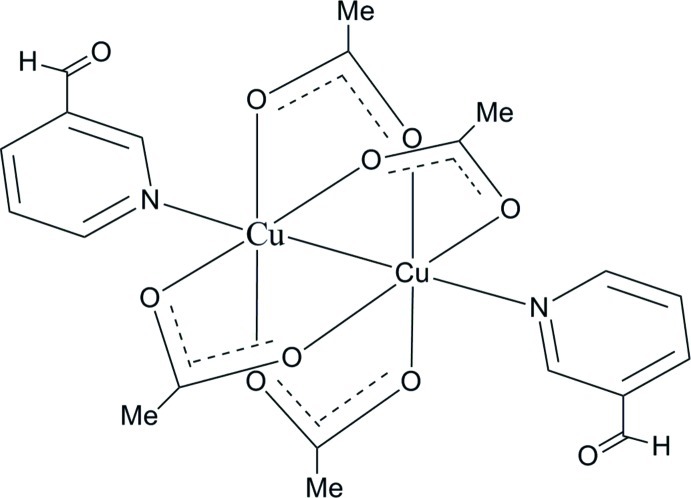


## Experimental

### 

#### Crystal data

[Cu_2_(C_2_H_3_O_2_)_4_(C_6_H_5_NO)]
*M* *_r_* = 577.48
Triclinic, 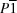

*a* = 7.4099 (6) Å
*b* = 8.4298 (7) Å
*c* = 10.0254 (8) Å
α = 100.353 (1)°
β = 108.975 (1)°
γ = 98.679 (1)°
*V* = 567.61 (8) Å^3^
*Z* = 1
Mo *K*α radiation
μ = 1.93 mm^−1^
*T* = 100 K
0.48 × 0.21 × 0.17 mm

#### Data collection

Bruker SMART CCD area-detector diffractometer
Absorption correction: multi-scan (*SADABS*; Sheldrick, 1996 ▶) *T*_min_ = 0.448, *T*_max_ = 0.720
4118 measured reflections
1980 independent reflections
1921 reflections with *I* > 2σ(*I*)
*R*_int_ = 0.018

#### Refinement

*R*[*F* ^2^ > 2σ(*F* ^2^)] = 0.027
*wR*(*F* ^2^) = 0.068
*S* = 1.10
1980 reflections
156 parameters
H-atom parameters constrained
Δρ_max_ = 0.41 e Å^−3^
Δρ_min_ = −0.36 e Å^−3^

### 

Data collection: *SMART* (Bruker, 2000 ▶); cell refinement: *SAINT-Plus-NT* (Bruker, 2001 ▶); data reduction: *SAINT-Plus-NT*; program(s) used to solve structure: *SHELXS97* (Sheldrick, 2008 ▶); program(s) used to refine structure: *SHELXL97* (Sheldrick, 2008 ▶); molecular graphics: *ORTEP-3* (Farrugia, 2012 ▶) and *Mercury* (Macrae *et al.* 2008 ▶); software used to prepare material for publication: *publCIF* (Westrip, 2010 ▶).

## Supplementary Material

Crystal structure: contains datablock(s) I, New_Global_Publ_Block. DOI: 10.1107/S1600536812041074/su2505sup1.cif

Structure factors: contains datablock(s) I. DOI: 10.1107/S1600536812041074/su2505Isup2.hkl

Additional supplementary materials:  crystallographic information; 3D view; checkCIF report

## Figures and Tables

**Table 1:** Hydrogen-bond geometry (Å, °)

*D*—H⋯*A*	*D*—H	H⋯*A*	*D*⋯*A*	*D*—H⋯*A*
C3—H3⋯O1^i^	0.93	2.43	3.262 (4)	148
C6—H6⋯O3^ii^	0.93	2.56	3.449 (4)	159
C8—H8*B*⋯O3^iii^	0.96	2.60	3.542 (3)	168
C10—H10*C*⋯O2^iv^	0.96	2.48	3.420 (4)	167

Symmetry codes: (i) 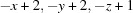; (ii) 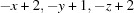; (iii) 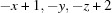; (iv) 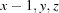.

**Fig. 1.**
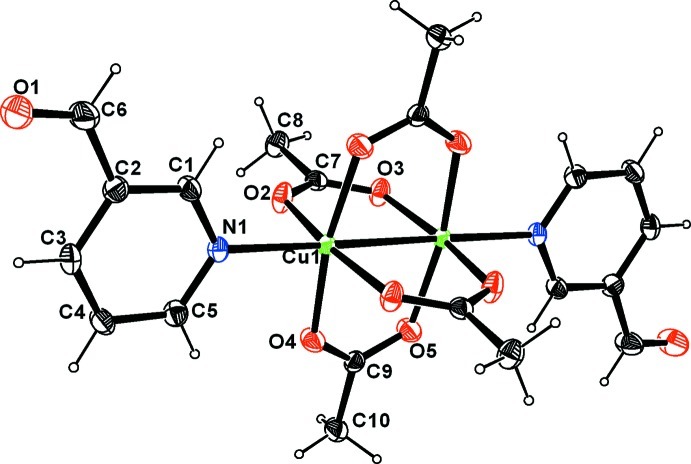
The molecular structure of the title complex, showing the atom-labelling scheme. The displacement ellipsoids are drawn at the 50% probability level. The unlabelled atoms are related by the symmetry code: -x+1, -y+1, -z+2.

**Fig. 2.**
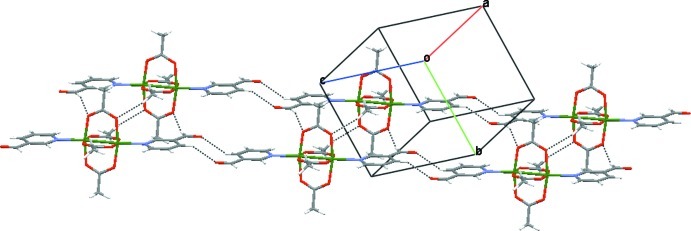
Perspective view of a fragment of the two-dimensional supramolecular network with the C—H···O hydrogen bonds shown as dashed lines.

## supplementary crystallographic information

### Comment

Bridged binuclear copper(II) complexes have been the subject of continuing interest because of their magneto-structural properties. Pyridine ligands have the potential to be used in the synthesis of supramolecular materials, particularly transition metal coordination polymers. Herein, we report on the synthesis and crystal structure of the new binuclear Cu_2_(OAc)_4_L_2_ paddle-wheel complex with L = 3-pyridincarboxaldehyde.

The title complex (Fig. 1) is structurally similar to paddle-wheel structures of other Cu_2_(OAc)_4_*L*_2_ complexes (Aakeröy *et al.*, 2003; Fairuz *et al.*, 2011; Seco *et al.*, 2004; Sieroń, 2004; Trivedi *et al.*, 2011). The binuclear molecule lies about an inversion center. Attached to the Cu_2_(OAc)_4_ core unit there are two apical pyridine groups from 3-pyridincarboxaldehyde ligands. The Cu—O distances [which vary from 1.9625 (19) - 1.9692 (17) Å], the Cu1—N1 distance [2.189 (2) Å], and the corresponding bond angles are consistent with the structurally similar Cu^II^complexes mentioned above. Although the Cu1—Cu1^i^ separation of 2.6134 (6) Å [symmetry code: (i) -x+1, -y+1, -z+2] is towards the lower limit, it is within the range of values reported for other Cu^II^ paddle-wheel structures (Sieroń, 2004; Asem *et al.*, 2011).

In the crystal, the complex molecules are linked by C—H···O hydrogen bonds to form two-dimensional networks lying parallel to the ab plane (Table 1 and Fig. 2). These networks are linked via C-H···O hydrogen bonds involving inversion related 3-pyridincarboxaldehyde ligands forming a three dimensional supramolecular architecture (Table 1).

### Experimental

A mixture of 3-pyridincarboxaldehyde (0.05 g, 0.466 mmol) and Cu(CH_3_COO)_2_H_2_O (0.093 g, 0.466 mmol) dissolved in methanol (5 ml) was stirred for 2 h at room temperature to give a blue solution. After one week, blue crystals suitable for X-ray diffraction analysis had been formed, which were collected by filtration [Yield: 65%]. Spectroscopic and TGA data are given in the archived CIF.

### Refinement

The C-bound H-atoms were included in calculated positions and treated as riding atoms: C-H = 0.93 and 096 Å for CH and CH_3_ H-atoms, respectively, with U_iso_(H) = k × U_eq_(parent C-atom), where k = 1.5 for CH_3_ H-atoms and = 1.2 for other H-atoms.

### Crystal data

**Table:**

[Cu_2_(C_2_H_3_O_2_)_4_(C_6_H_5_NO)]	*Z* = 1
*M**_r_* = 577.48	*F*(000) = 294
Triclinic, *P*1	*D*_x_ = 1.689 Mg m^−^^3^
Hall symbol: -P 1	Mo *K*α radiation, λ = 0.71073 Å
*a* = 7.4099 (6) Å	Cell parameters from 3727 reflections
*b* = 8.4298 (7) Å	θ = 2.5–28.3°
*c* = 10.0254 (8) Å	µ = 1.93 mm^−^^1^
α = 100.353 (1)°	*T* = 100 K
β = 108.975 (1)°	Rectangular prism, blue
γ = 98.679 (1)°	0.48 × 0.21 × 0.17 mm
*V* = 567.61 (8) Å^3^	

### Data collection

**Table:**

Bruker SMART CCD area-detector diffractometer	1980 independent reflections
Radiation source: fine-focus sealed tube	1921 reflections with *I* > 2σ(*I*)
Graphite monochromator	*R*_int_ = 0.018
phi and ω scans	θ_max_ = 25.0°, θ_min_ = 2.2°
Absorption correction: multi-scan (*SADABS*; Sheldrick, 1996)	*h* = −8→8
*T*_min_ = 0.448, *T*_max_ = 0.720	*k* = −8→10
4118 measured reflections	*l* = −11→11

### Refinement

**Table:**

Refinement on *F*^2^	Primary atom site location: structure-invariant direct methods
Least-squares matrix: full	Secondary atom site location: difference Fourier map
*R*[*F*^2^ > 2σ(*F*^2^)] = 0.027	Hydrogen site location: inferred from neighbouring sites
*wR*(*F*^2^) = 0.068	H-atom parameters constrained
*S* = 1.10	*w* = 1/[σ^2^(*F*_o_^2^) + (0.0297*P*)^2^ + 0.5378*P*] where *P* = (*F*_o_^2^ + 2*F*_c_^2^)/3
1980 reflections	(Δ/σ)_max_ = 0.001
156 parameters	Δρ_max_ = 0.41 e Å^−^^3^
0 restraints	Δρ_min_ = −0.36 e Å^−^^3^

### Special details

**Table:**

Experimental. Spectroscopic and TGA data for the title compound: IR (KBr, cm^-1^): 3273, 3076, 2935, 2871, 1705, 1615, 1440, 1218, 1035, 690; TGA Calcd. for 2(C_6_H_5_NO): 37.07. Found: 37.53% (303 - 473 K).
Geometry. Bond distances, angles etc. have been calculated using the rounded fractional coordinates. All su's are estimated from the variances of the (full) variance-covariance matrix. The cell esds are taken into account in the estimation of distances, angles and torsion angles
Refinement. Refinement of *F*^2^ against ALL reflections. The weighted *R*-factor *wR* and goodness of fit *S* are based on *F*^2^, conventional *R*-factors *R* are based on *F*, with *F* set to zero for negative *F*^2^. The threshold expression of *F*^2^ > σ(*F*^2^) is used only for calculating *R*-factors(gt) *etc*. and is not relevant to the choice of reflections for refinement. *R*-factors based on *F*^2^ are statistically about twice as large as those based on *F*, and *R*- factors based on ALL data will be even larger.

### Fractional atomic coordinates and isotropic or equivalent isotropic displacement parameters (Å^2^)

**Table:**

	*x*	*y*	*z*	*U*_iso_*/*U*_eq_	
Cu1	0.56063 (4)	0.57222 (3)	0.91251 (3)	0.0137 (1)	
O1	1.1945 (3)	0.9402 (3)	0.6523 (2)	0.0382 (7)	
O2	0.6188 (3)	0.3569 (2)	0.85203 (19)	0.0232 (5)	
O3	0.5192 (3)	0.2340 (2)	1.00330 (18)	0.0216 (5)	
O4	0.2879 (2)	0.4870 (2)	0.77698 (18)	0.0235 (5)	
O5	0.1855 (2)	0.3580 (2)	0.92442 (18)	0.0243 (5)	
N1	0.6613 (3)	0.6761 (2)	0.7573 (2)	0.0155 (6)	
C1	0.8501 (4)	0.7380 (3)	0.7858 (2)	0.0192 (7)	
C2	0.9167 (4)	0.8063 (3)	0.6899 (3)	0.0203 (7)	
C3	0.7811 (4)	0.8086 (3)	0.5568 (3)	0.0215 (8)	
C4	0.5870 (4)	0.7427 (3)	0.5258 (3)	0.0223 (7)	
C5	0.5325 (4)	0.6786 (3)	0.6284 (3)	0.0198 (7)	
C6	1.1288 (4)	0.8746 (4)	0.7290 (3)	0.0306 (9)	
C7	0.5933 (3)	0.2365 (3)	0.9070 (2)	0.0172 (7)	
C8	0.6587 (4)	0.0847 (3)	0.8539 (3)	0.0230 (8)	
C9	0.1593 (3)	0.3988 (3)	0.8058 (2)	0.0168 (7)	
C10	−0.0418 (4)	0.3371 (3)	0.6896 (3)	0.0242 (8)	
H1	0.94130	0.73520	0.87420	0.0230*	
H3	0.82120	0.85370	0.49020	0.0260*	
H4	0.49320	0.74120	0.43710	0.0270*	
H5	0.40040	0.63520	0.60690	0.0240*	
H6	1.21540	0.86630	0.81720	0.0370*	
H8A	0.58970	0.04310	0.75100	0.0340*	
H8B	0.63120	0.00150	0.90290	0.0340*	
H8C	0.79690	0.11290	0.87380	0.0340*	
H10A	−0.06390	0.22020	0.65090	0.0360*	
H10B	−0.05110	0.39310	0.61300	0.0360*	
H10C	−0.13850	0.35880	0.73070	0.0360*	

### Atomic displacement parameters (Å^2^)

**Table:**

	*U*^11^	*U*^22^	*U*^33^	*U*^12^	*U*^13^	*U*^23^
Cu1	0.0144 (2)	0.0153 (2)	0.0134 (2)	0.0035 (1)	0.0066 (1)	0.0051 (1)
O1	0.0245 (10)	0.0576 (14)	0.0370 (11)	0.0009 (9)	0.0121 (9)	0.0276 (10)
O2	0.0356 (10)	0.0191 (9)	0.0269 (9)	0.0118 (8)	0.0217 (8)	0.0106 (7)
O3	0.0309 (10)	0.0186 (9)	0.0244 (9)	0.0100 (7)	0.0174 (8)	0.0096 (7)
O4	0.0165 (9)	0.0326 (10)	0.0188 (8)	−0.0007 (8)	0.0039 (7)	0.0096 (7)
O5	0.0168 (9)	0.0343 (10)	0.0200 (9)	0.0000 (7)	0.0050 (7)	0.0103 (8)
N1	0.0195 (10)	0.0129 (10)	0.0157 (9)	0.0028 (8)	0.0083 (8)	0.0042 (8)
C1	0.0222 (13)	0.0214 (12)	0.0162 (11)	0.0070 (10)	0.0069 (10)	0.0085 (9)
C2	0.0211 (13)	0.0213 (13)	0.0226 (12)	0.0068 (10)	0.0102 (10)	0.0096 (10)
C3	0.0266 (14)	0.0243 (13)	0.0180 (12)	0.0052 (11)	0.0125 (11)	0.0079 (10)
C4	0.0231 (13)	0.0276 (14)	0.0146 (11)	0.0042 (11)	0.0042 (10)	0.0072 (10)
C5	0.0194 (12)	0.0199 (12)	0.0190 (12)	0.0018 (10)	0.0072 (10)	0.0042 (10)
C6	0.0220 (14)	0.0439 (17)	0.0291 (14)	0.0053 (12)	0.0082 (12)	0.0202 (13)
C7	0.0139 (11)	0.0195 (12)	0.0154 (11)	0.0018 (9)	0.0029 (9)	0.0033 (9)
C8	0.0263 (14)	0.0195 (13)	0.0260 (13)	0.0067 (10)	0.0128 (11)	0.0051 (10)
C9	0.0161 (12)	0.0176 (12)	0.0166 (11)	0.0048 (10)	0.0068 (10)	0.0018 (9)
C10	0.0187 (13)	0.0334 (15)	0.0181 (12)	0.0028 (11)	0.0053 (10)	0.0053 (10)

### Geometric parameters (Å, º)

**Table:**

Cu1—O2	1.9643 (19)	C3—C4	1.371 (4)
Cu1—O4	1.9677 (17)	C4—C5	1.385 (4)
Cu1—N1	2.189 (2)	C7—C8	1.505 (4)
Cu1—O3^i^	1.9625 (19)	C9—C10	1.506 (4)
Cu1—O5^i^	1.9692 (17)	C1—H1	0.9300
O1—C6	1.204 (4)	C3—H3	0.9300
O2—C7	1.257 (3)	C4—H4	0.9300
O3—C7	1.258 (3)	C5—H5	0.9300
O4—C9	1.260 (3)	C6—H6	0.9300
O5—C9	1.260 (3)	C8—H8A	0.9600
N1—C1	1.333 (4)	C8—H8B	0.9600
N1—C5	1.340 (3)	C8—H8C	0.9600
C1—C2	1.386 (4)	C10—H10A	0.9600
C2—C3	1.391 (4)	C10—H10B	0.9600
C2—C6	1.483 (4)	C10—H10C	0.9600
			
O2—Cu1—O4	89.70 (8)	O3—C7—C8	117.9 (2)
O2—Cu1—N1	92.99 (8)	O4—C9—O5	125.2 (2)
O2—Cu1—O3^i^	168.80 (8)	O4—C9—C10	117.59 (19)
O2—Cu1—O5^i^	90.47 (8)	O5—C9—C10	117.3 (2)
O4—Cu1—N1	94.72 (7)	N1—C1—H1	118.00
O3^i^—Cu1—O4	88.36 (8)	C2—C1—H1	119.00
O4—Cu1—O5^i^	168.89 (7)	C2—C3—H3	121.00
O3^i^—Cu1—N1	98.16 (7)	C4—C3—H3	121.00
O5^i^—Cu1—N1	96.36 (7)	C3—C4—H4	120.00
O3^i^—Cu1—O5^i^	89.32 (8)	C5—C4—H4	120.00
Cu1—O2—C7	124.41 (17)	N1—C5—H5	118.00
Cu1^i^—O3—C7	121.63 (16)	C4—C5—H5	119.00
Cu1—O4—C9	123.33 (14)	O1—C6—H6	118.00
Cu1^i^—O5—C9	122.48 (15)	C2—C6—H6	118.00
Cu1—N1—C1	122.12 (14)	C7—C8—H8A	109.00
Cu1—N1—C5	120.33 (19)	C7—C8—H8B	110.00
C1—N1—C5	117.6 (2)	C7—C8—H8C	109.00
N1—C1—C2	123.1 (2)	H8A—C8—H8B	109.00
C1—C2—C3	118.7 (3)	H8A—C8—H8C	109.00
C1—C2—C6	120.3 (3)	H8B—C8—H8C	110.00
C3—C2—C6	121.0 (3)	C9—C10—H10A	109.00
C2—C3—C4	118.6 (3)	C9—C10—H10B	109.00
C3—C4—C5	119.1 (3)	C9—C10—H10C	110.00
N1—C5—C4	123.1 (3)	H10A—C10—H10B	109.00
O1—C6—C2	123.3 (3)	H10A—C10—H10C	110.00
O2—C7—O3	125.1 (2)	H10B—C10—H10C	109.00
O2—C7—C8	117.1 (2)		
			
O2—Cu1—N1—C1	−82.47 (19)	Cu1—O2—C7—C8	−176.00 (17)
O2—Cu1—N1—C5	97.24 (19)	Cu1—O2—C7—O3	3.4 (3)
O4—Cu1—N1—C1	−172.41 (18)	Cu1^i^—O3—C7—O2	−2.2 (3)
O4—Cu1—N1—C5	7.30 (19)	Cu1^i^—O3—C7—C8	177.20 (17)
O3^i^—Cu1—N1—C1	98.57 (19)	Cu1—O4—C9—O5	2.2 (3)
O3^i^—Cu1—N1—C5	−81.72 (19)	Cu1—O4—C9—C10	−177.83 (16)
O5^i^—Cu1—N1—C1	8.34 (19)	Cu1^i^—O5—C9—O4	−0.1 (3)
O5^i^—Cu1—N1—C5	−171.95 (18)	Cu1^i^—O5—C9—C10	179.91 (17)
O4—Cu1—O2—C7	−86.4 (2)	Cu1—N1—C1—C2	−179.13 (19)
N1—Cu1—O2—C7	178.9 (2)	C5—N1—C1—C2	1.2 (4)
O5^i^—Cu1—O2—C7	82.5 (2)	Cu1—N1—C5—C4	179.93 (19)
O5—Cu1^i^—O3—C7	85.16 (19)	C1—N1—C5—C4	−0.4 (4)
O4^i^—Cu1^i^—O3—C7	−83.97 (19)	N1—C1—C2—C6	179.4 (2)
N1^i^—Cu1^i^—O3—C7	−178.51 (18)	N1—C1—C2—C3	−0.9 (4)
O2—Cu1—O4—C9	80.85 (19)	C1—C2—C6—O1	−177.5 (3)
N1—Cu1—O4—C9	173.83 (18)	C6—C2—C3—C4	179.5 (3)
O3^i^—Cu1—O4—C9	−88.12 (19)	C1—C2—C3—C4	−0.2 (4)
O3—Cu1^i^—O5—C9	−86.94 (19)	C3—C2—C6—O1	2.8 (5)
O2^i^—Cu1^i^—O5—C9	81.86 (19)	C2—C3—C4—C5	0.9 (4)
N1^i^—Cu1^i^—O5—C9	174.92 (18)	C3—C4—C5—N1	−0.7 (4)

Symmetry code: (i) −*x*+1, −*y*+1, −*z*+2.

### Hydrogen-bond geometry (Å, º)

**Table:**

*D*—H···*A*	*D*—H	H···*A*	*D*···*A*	*D*—H···*A*
C3—H3···O1^ii^	0.93	2.43	3.262 (4)	148
C6—H6···O3^iii^	0.93	2.56	3.449 (4)	159
C8—H8*B*···O3^iv^	0.96	2.60	3.542 (3)	168
C10—H10*C*···O2^v^	0.96	2.48	3.420 (4)	167

Symmetry codes: (ii) −*x*+2, −*y*+2, −*z*+1; (iii) −*x*+2, −*y*+1, −*z*+2; (iv) −*x*+1, −*y*, −*z*+2; (v) *x*−1, *y*, *z*.
